# Editorial: Emerging pathogens and contaminants in the environment: human health risks, exposure pathways and epidemiological outcomes

**DOI:** 10.3389/fmicb.2026.1787675

**Published:** 2026-02-02

**Authors:** Anthony A. Adegoke, Olayinka A. Aiyegoro, Collins N. Ateba, Sunday Oyedemi

**Affiliations:** 1 Department of Microbiology, Faculty of Science, University of Uyo, Uyo, Nigeria; 2 Department of Community Health Studies, Faculty of Health Sciences, Durban University of Technology, Durban, KwaZulu-Natal, South Africa; 3 Research Unit for Environmental Sciences and Management, North West University, Potchefstroom, North West, South Africa; 4 Faculty of Agriculture and Natural Sciences, School of Biology and Environmental Sciences, University of Mpumalanga, Mbombela, South Africa; 5 School of Science and Technology, Nottingham Trent University, Nottingham, United Kingdom

**Keywords:** emerging pathogens, environmental exposure, epidemiology, human health, risks

## 1 Conceptualization

After embarking on a Nigerian-based research project under the National Research Fund (NRF) of the TETFUND, Project Code TETF/DR&D CE/NRF/2020/SETI/99/VOL.1, this new expansion was birthed. The scope of the previously completed Research Topic was broadened from water-based factors to the impact of the general environment in the conveyance of pathogens and contaminants, together with associated human risks and epidemiological outcomes. The research work we embarked on, which propelled this extension centered on the risk assessment of *Listeria monocytogenes* in food grown using organic fertilizers.

## 2 Emerging pathogens and contaminants in the environment

The intensification of human activity introduces novel microbial pathogens and anthropogenic contaminants (Pruden et al., 2006), disrupting environmental ecosystems and exerting selective pressures that favor microorganisms with adaptive traits such as antimicrobial resistance or pollutant degradation (Faleye et al., 2017; Adegoke et al., 2020). These stressors are disseminated through interconnected aquatic and terrestrial systems via runoff, leaching, and infrastructure failures, facilitating widespread distribution. Human exposure occurs through ingestion, dermal contact, and inhalation, with health outcomes shaped by exposure intensity, duration, and concentration (Adegoke et al., 2017; Faleye et al., 2017).

Alongside beneficial microbiota, environmental systems harbor opportunistic and pathogenic species, whose proliferation is often linked to increased human-animal interactions and habitat encroachment. These dynamics foster the cross-species transmission of emerging and re-emerging pathogens, posing significant public health threats (Adegoke et al., 2020). Concurrently, industrial, agricultural, and urban waste streams introduce trace-level chemical contaminants, including carcinogens and endocrine disruptors, which can drive ecological disruption and select for resistant microbes. Effective mitigation requires robust identification of pollutants and pathogens, comprehensive exposure assessment, and a clear understanding of their epidemiological impacts to inform sustainable environmental and public health strategies.

## 3 Evidence-based evolving global dynamic microbial threat patterns

In many regions, well-known pathogens continue to exert a significant toll through persistent environmental contamination. Research from Northwest Ethiopia, for example, reveals a 39.5% prevalence of intestinal helminths like *Ascaris lumbricoides* among schoolchildren (Wube et al.). Key exposure pathways were linked to poor hygiene and consumption of contaminated food, with lower maternal education identified as a major risk factor. Similarly, water and soil serve as reservoirs for severe bacterial infections. Work on *Burkholderia pseudomallei* demonstrates that this soil-dwelling bacterium produces 2-alkylquinolone metabolites, which serve a dual function: enhancing its environmental fitness against competing microbes and acting as critical virulence factors during human melioidosis infection (Mou et al.).

The re-emergence of vaccine-preventable diseases also signals epidemiological shifts. Brazil experienced a dramatic 344-fold surge in pertussis cases from 2023 to 2024 (Sansone et al.). This respiratory transmission led to increased hospitalizations, particularly among infants, highlighting gaps in population immunity. Meanwhile, leptospirosis remains a major global zoonosis. According to a recent systematic review, transmission occurs through water contaminated by infected animal urine, with disease severity ranging from mild illness to fatal organ failure, especially in tropical regions (Wang et al.).

Environmental control measure is imperative for respiratory viruses. Based on the work of Guo et al., the evolution of the SARS-CoV-2 pathogen is critically shaped by environmental control measures. Their model shows that non-pharmaceutical interventions (NPIs) select for variants with higher transmissibility and immune evasion, directly linking human interventions to viral evolutionary pathways and the risk of recurrent epidemics, underscoring the need for adaptive surveillance.

Changes in diarrheal disease etiology further illustrate this dynamic environment. In a cholera-endemic area of India, Enterotoxigenic *Escherichia coli* (ETEC) emerged as the dominant pathogen, responsible for 20.3% of cases (Khuntia et al.). This shift underscores the persistent health risk from the fecal-oral route via contaminated food and water.

## 4 Breaking species barriers and global dissemination

The traditional paradigm of host-specific pathogens is being challenged. Serological evidence indicates that porcine norovirus GIL.11 can cross species barriers (Xu et al.). Antibodies were detected not only in pigs but also in humans (15.2%) and various wildlife, with bats showing the highest seroprevalence (Xu et al.). Similarly, the epidemiology of Q fever (*Coxiella burnetii*) is more complex than previously thought. Studies in Latin America document high infection rates not just among livestock workers but also in urban, incarcerated, and indigenous populations, suggesting significant environmental or airborne transmission (França).

Shi et al. characterized the 2023 human monkeypox outbreak in Zhejiang, China, identifying local transmission, high prevalence in Men who have Sex with Men (MSM), and clinical profiles, thereby informing public health surveillance. Also, 2022–2023 global monkeypox outbreak exemplifies the rapid adaptation of a zoonotic virus. Malla and Saleh review how Monkeypox Virus (MPXV) transitioned from sporadic spillover events to sustained human-to-human transmission worldwide, exposing critical gaps in global surveillance and vaccine equity.

**Figure 1:**
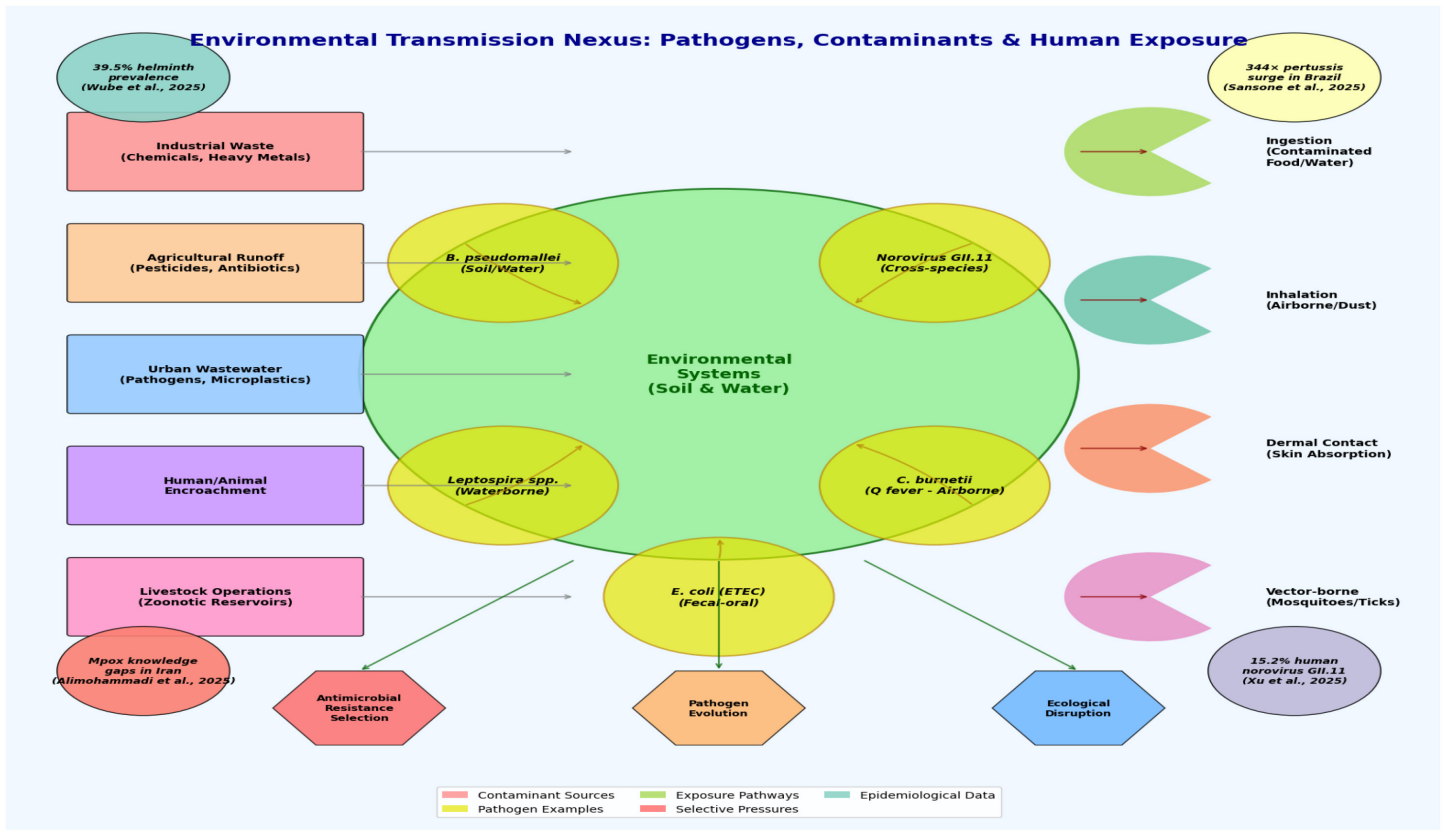
Conceptual framework illustrating the complex interactions between environmental contaminants, emerging pathogens, and human exposure pathways [Arrows indicate flow and transmission routes].

Figure 1 illustrates the conceptual framework, which shows the complex interactions between environmental contaminants, emerging pathogens, and human exposure pathways. This depicts the overall potential flow in the environment.

## 5 Knowledge gaps and toxins' contaminants

Public and professional awareness remains a cornerstone of effective outbreak response. Alimohamadi et al. reported significant knowledge deficits about mpox among Iranian university students and staff, with social media being a primary, and often unreliable, source of information. This reliance heightens the risk of misinformation undermining public health efforts.

Beyond biological agents, environmental biochemical contaminants present insidious exposure risks. Investigations into occupational settings, such as a waste management study in Norway, have highlighted human exposure to mycotoxins (Martins et al.). These fungal metabolites represent an important class of environmental contaminants requiring vigilant exposure assessment.

## References

[B1] Adegoke A. A. Fatunla O. K. Okoh A. I. (2020). Critical threat associated with carbapenem-resistant gram-negative bacteria: prioritizing water matrices in addressing total antibiotic resistance. Ann. Microbiol. 70:43. doi: 10.1186/s13213-020-01579-4

[B2] Adegoke A. A. Stenström T. A. Okoh A. I. (2017). *Stenotrophomonas maltophilia* as an emerging ubiquitous pathogen: looking beyond contemporary antibiotic therapy. Front. Microbiol. 8:2276. doi: 10.3389/fmicb.2017.02276 29250041 PMC5714879

[B3] Faleye C. A. Adegoke A. A. Ramluckan K. Bux F. Stenström T. A. (2017). Identification of antibiotics in wastewater: current state of extraction protocol and future perspectives. J. Water Health 15, 982–1003. doi: 10.2166/wh.2017.097 29215361

[B4] Pruden A. Pei R. Storteboom H. Carlson K. H. (2006). Antibiotic resistance genes as emerging contaminants: studies in northern Colorado. Environ. Sci. Technol. 40, 7445–7450. doi: 10.1021/es060413l 17181002

